# Nematicidal activity of sweet annie and garden cress nano-formulations and their impact on the vegetative growth and fruit quality of tomato plants

**DOI:** 10.1038/s41598-022-26819-2

**Published:** 2022-12-24

**Authors:** Ayman A. Mohammad, Heba M. Amer, Sameh M. El-Sawy, Dalia A. Youssef, Shaimaa A. Nour, Gaziea M. Soliman

**Affiliations:** 1 Food Technology Department, National Research Centre, Dokki, 12622 Cairo Egypt; 2 Medicinal and Aromatic Plants Research Department, National Research Centre, Dokki, 12622 Cairo Egypt; 3 Vegetable Research Department, National Research Centre, Dokki, 12622 Cairo Egypt; 4 Pests and Plant Protection Department, National Research Centre, Dokki, 12622 Cairo Egypt; 5 Chemistry of Natural and Microbial Products Department, National Research Centre, Dokki, 12622 Cairo Egypt; 6 Plant Pathology Department, Nematology Unit, National Research Centre, Dokki, 12622 Cairo Egypt

**Keywords:** Biological techniques, Nanoscience and technology

## Abstract

Root-knot nematode is one of the major problems that face the agricultural production of several vegetable crops. Chemical nematicides have been banned because of their healthy and environmental undesirable attributes. So, this study aimed to evaluate the potential use of sweet annie (*Artimisia annua*) and garden cress (*Lepidium sativum*) as green routes for the development of effective and eco-friendly alternative nematicides. Nematicidal activity of sweet annie and garden cress aqueous extracts (500 g/L) in the original and nano-forms were evaluated against *Meloidogyne incognita* in tomato planted in infected soil under greenhouse conditions. Nineteen phenolic compounds were identified in *A. annua* extract, which was dominated by chlorogenic acid (5059 µg/100 mL), while 11 compounds were identified in *L. sativum* extract, that dominated by *p*-hydroxybenzoic acid (3206 μg/100 mL). Nano-particles were characterized with smooth surface, spherical shape and small size (50–100 nm). Under laboratory, the nano-formulations showed mortality percentage of *M. incognita* J_2_ greater than the original extract from. Vegetative growth parameters of tomato plants treated with *A. annua* and *L. sativum* extracts significantly improved compared to the control plants. Also, biochemical analysis revealed that the extracts were able to induce tomato plants towards the accumulation of phenolic compounds and increasing the activity of defensive enzymes (protease, polyphenol oxidase and chitinase) resulting in systemic resistance. Regarding tomato fruits yield and quality, the studied treatments significantly improved the yield and physicochemical parameters of tomato fruits in terms of fruit weight, diameter, TSS, pH, lycopene content and color attributes gaining higher sensorial acceptance by the panelist. Generally, both extracts represent promising nematicide alternatives and have potential use in crop management. The nano-form of *A. annua* extract outperformed the nematicidal activity of other studied treatments.

## Introduction

Tomato (*Solanum lycopersicum*) is a popular vegetable crop and widely consumed all over the world. Egypt ranks sixth in the production of tomato^1^. The tomato agricultural production faces several major problems which lead to low crop yield. Root-knot nematodes (*Meloidogyne* spp.) are among the most destructive agricultural pests globally, particularly vegetables, that cause serious yield losses in tomato production. *M. incognita* is one of the most economically important nematode species since it can attack the roots of over 3000 agricultural crops^2,3^. Tomato yields had reduced by about 25.0–49.0% by *M. incognita* attack^4^.

The increased environmental concern, as well as the recent prohibition on numerous nematicides, has necessitated a reduction in chemical nematicides and the creation of non-chemical alternatives^5^. Furthermore, the search for effective, ecologically friendly, and safe alternative controls has accelerated. Because the use of nematicides is becoming unaffordable in many countries due to their negative repercussions, green-nanoformulation for the management of plant-parasitic nematodes using nanoparticles can be a viable option^6^.

Nanotechnology provides innovative agricultural practices and revolutionizes the existing pest management applications^7,8^. The agricultural nanotechnology provides the potential of new nematicides generation. Nano-capsules are vesicular systems in which a single polymeric membrane (wall material) covers specific substances that have been solubilized in an aqueous or oil core^9^. Nano-capsules have longer period of effective action in a single use of any nematicides, which effectively reduce the level of nematode infestation.

Phytochemicals of several higher plants offer attractive potential as nematicidal agents for crop protection^10^. Nematicidal extracts or phytochemicals are environment friendly^11^. In this concern, *Artemisia* spp. has produced about 1000 biodynamic chemicals and several types of secondary metabolites including, phenolic, furans and flavonoids^12^. Also, the herb of *L. sativum* includes volatile essential fragrant oils, ascorbic acid, flavonoids, and isothiocynate glycosides^13,14^. The Artemisia species extracts have active compounds that reduce the infectivity potential of root-knot nematodes, and kill the infective juveniles^15–17^. The aqueous extracts of dried plant shoots and dried aerial parts effectively prevented juvenile mobility, egg hatching and killed *M. incognita* second-stage juveniles^15,18^.

Induced resistance and reducing diseases infection in plants have been related to a large number of enzymes such as polyphenol oxidase, β-1.3-glucanase and chitinase^19^. These enzymes play an important role in the degradation of pathogen cell walls, releasing chemicals that act as elicitors in the early stages of phytoalexin and formation of phenolic compounds and other pathogenesis proteins or metabolites related plant defense mechanisms^20–22^.

Based on the aforementioned facts, the present study was designed to synthesize nanoparticles via a green route using *A. annua* and *L. sativum* aqueous extracts, with the goal of identifying new eco-friendly nematicides that could be used to reduce nematode infection and increase plant yield. The nematicidal activity on juveniles mortality and tomato plant infected with *M. incognita* were evaluated. The vegetative growth parameters and the biochemical alteration changes in the host during infection and bio-control of *M. incognita* as well as the yield and tomato fruit quality were monitored.

## Methods

### Preparation of root-knot nematode culture

A pure nematode population used in laboratory bioassay were obtained from tomato plants grown in screen house on in a 1:1 mixture of clay and sand maintained at the Plant Pathology Department and identified as *Meloidogyne incognita* by Light microscopic examinations of perennial pattern of female^23^. Nematode eggs were extracted from the *M. incognita*-infected tomato roots through stirring of washed tomato roots pieces in NaOCl (0.5%) for 12 min according to the method of Hussey and Barker^24^as described by Gómez-González et al.^25^. The eggs were hatched out in water for 48 h at room temperature (27 ± 2 °C) using the modified Baermann plates according to Barker^26^ and the second larval stages (J_2_s) were used immediately for juvenile mortality test.

### Preparation of Aqueous plant extracts

Sweet annie (*Artimisia annua* L.) and Garden cress (*Lepidium sativum* L.) seeds were obtained from Agriculture Research Center, Giza, Egypt. Seeds were planted in natural outdoor micro-plots in the farm of the National Research Center, and the fully grown plants were harvested at floral initiation. The obtained plants were identified and authenticated by Mrs. Therese Labib, Consultant of Plant Taxonomy at the Ministry of Agriculture and Ex-director of the Orman Botanical Garden, Giza, Egypt. The aerial plant parts were shade-dried at room temperature. 500 g of dried *A. annua* and *L. sativum* coarse powders were separately macerated in one liter of warm water (30 °C) for 16 h at room temperature (25 ± 3 °C) with occasional stirring, and then filtered through a Whatman No. 54 filter paper. The obtained extracts were considered original and marked as A_500_ and L_500_, respectively. Additional dilutions (A_250_, A_125_, L_250_ and L_125_) were prepared from the original extracts.

### Estimation of bioactive compounds

Phenolic compounds of *A. annua* and *L. sativum* extracts were estimated using HP 1090 M Series II HPLC system equipped with diode array detector (EC; Esa Inc., USA) and HP 3D ChemStation computer program. The analytical column was Eclipse XDB-C18 (150 × 4.6 µm; 5 µm) with a C18 guard column (Phenomenex, Torrance, CA). Gradient elution was employed with a mobile phase consisting of 50 mM H_3_PO_4_, pH 2.5 (solution A) and acetonitrile (solution B). The flow rate was kept at 1 mL/min, column oven temperature was set at 35 °C and diode array detector wavelengths were 280 and 330 nm. The injection volumes were 10 µL of the standards mixture and sample extracts. Quantification, based on peak area, was calculated using standard calibration curves of 20 compounds. The samples were analyzed in duplicate and the obtained results expressed in μg/100 mL.

### Preparation of nano-emulsions

Nano-emulsions of medicinal plant extracts (*A. annua* and *L. sativum*) were prepared by the micro mini-emulsion polymerization method using poly ethylene glycol (PEG) as described by Zhang et al.^27^. The extract suspension was dropwised in polyethylene glycol solution (3%) in a ratio of 1:1 (v/v) under continuous mechanical stirring at room temperature. The suspension of the extract was sonicated for 60 min using ultrasonic cleaner set, model WUC-DO3H 290 W and 60 Hz, and then sonicated for 3 min using a high energy ultra-sonication probe (model VCX750, 750 W, 20 kHz). The loaded nano-capsule suspension was equilibrated overnight. The prepared nano-emulsions were marked as NA_500_ and NL_500_ for *A. annua* and *L. sativum*, respectively. Additional dilutions (NA_250_, NA_125_, NL_250_ and NL_125_) were prepared.

### Transmission electron microscopy of nano-emulsion

The morphological shapes of prepared emulsions were tested with Transmission Electron Microscopy (TEM) (Jeol, JEM-2100). The prepared suspensions were diluted with distilled water and deposited onto a carbon-coated copper grid and then examined by magnification (20000X) and photographed.

### Estimation of nematicidal activity

#### Juvenile mortality

The nematicidal activity of *A. annua* (A_500_ and A_250_) and *L. sativum* (L_500_ and L_250_) extracts and their respective nano-formulations against *M. incognita* juveniles was tested, as follow: 4 mL from each solution were mixed with 1 mL containing 100 ± 5 juveniles of *M. incognita* in test tubes. The tubes were incubated at room temperature for 24 and 48 h in a completely randomized design. One mL from each tube was taken on a Hawksley counting slide and the numbers of dead juveniles were counted with the aid of light microscope. Also, after 48 h of exposure, the juveniles were washed by distilled water and transferred to aerated distilled water for 24 h and then the average percentages of nematode recoveries were determined. The mortality of juveniles was recorded after 24 and 48 h. Nematodes were considered alive if they moved or appeared as a winding shape and were considered dead if they did not move when probed with a fine needle^28^. All treatments were conducted in five biological replicates for two consecutive days and the average results were compared to negative control (water). The percentages of nematode mortality were calculated according to Abbott's Formula^29^ as follows:

$\mathrm{Juvenile mortality }(\mathrm{\%})\hspace{0.17em}=\hspace{0.17em}(m-n)/ (100-n)\hspace{0.17em}\times \hspace{0.17em}100,$where m and n indicate the percentages of mortality in treatments and control, respectively.

### Field experiment

Field experiment was carried out in a productive plastic greenhouse (naturally infested with *M. incognita*) at the Experimental and Production Station of National Research Centre, El-Noubaria region, Beheira Governorate**,** North of Egypt, during 2020 and 2021 seasons, Initial population densities of *M. incognita* were determined prior to planting time. Tomato seedlings (*Solanum lycopersicum* Mill. cv. CH7) were transplanted to the field experiment at September. The experiment was arranged in a randomized complete block design to 14 blocks. The first 2 blocks represent the negative control (untreated plants) and positive control (vydate, nematicide, treated plants with the recommended dose). Blocks no. 3–5 and 6–8 were treated with *A. annua* extracts (A_500_, A_250_ and A_125_) and their respective nano-formulation (NA_500_, NA_250_ and NA_125_), respectively. Similarly, blocks no. 9–11 and 12–14 were treated with *L. sativum* extracts (L_500_, L_250_ and L_125_) and their respective nano-formulation (NL_500_, NL_250_ and NL_125_), respectively. The extracts were added at 60 mL/plant by pouring the solution into holes made around the seedlings after 3 days of transferring. Different recommended agricultural practices for tomato plants were followed by the Ministry of Agric., Egypt. The effects of the prepared extracts and their nano-formulations on vegetative growth, biochemical parameters, flowering and fruit yield, fruit quality and root damage of tomato were evaluated.

### Vegetative growth parameters

Five plants were randomly chosen from each block at 65 days from transplanting date to determine the vegetative growth characteristics including plant length (cm), number of leaves per plant, number of branches per plant and fresh and dry weights of leaves per plant (g).

### Biochemical parameters of tomato plants

#### Protein estimation

Protein estimation was done according to Lowry et al.^30^ using Folin–Ciocalteu reagent and Bovine serum albumin as standard at 750 nm.

#### Total phenol contents determination

For assessing the total phenolic contents, 1 g fresh leaves of each treatment was homogenized in 10 mL of 80% methanol and agitated for 15 min at 70 °C. One mL of the extract was added to 5 mL distilled water plus 250 μL of 1 N Folin–Ciocalteu reagent. The absorbance was measured at 725 nm, and the amount of phenolic content was expressed as milligrams catechol equivalent per 100 g (mg CE/100 g)^31^.

#### Enzyme extraction

Plant samples (4 g) were cut and homogenized in 40 mL of phosphate buffer. The extract was then centrifuged at 10,000*g* for 10 min at 4 °C. The supernatant containing the crude enzyme extract was used for enzyme assay.

#### Polyphenol oxidase activity

The activity of polyphenol oxidase was determined according to Vamos-Vigyazo and Nadudvari-Marlcus^32^ using 0.5 mL of crude enzyme extract, 1 mL of catechol (0.05 M) as the substrate and 2.5 mL of phosphate buffer (0.1 M, pH 7.0). The absorbance was measured at 540 nm at a regular interval of 30 s.

#### Protease activity

Proteolytic activity on casein was determined according to the method described by Han and Damodaran^33^.

#### Chitinase activity

The exochitinase activity was determined using 0.1% of the synthetic substrate 4-nitrophenylN-acetyl-β-d-glucosaminide in 0.05 M phosphate buffer pH 6 according to Rustiguel et al.^34^.

### Flowering and fruit yield parameters

Flowering and fruit yield were determined in terms of number of clusters per plant, number of fruits per plant, fruit yield per plant (g) and total marketable yield (ton/fed.).

### Fruit quality parameters

Random samples of fruits were taken from each experimental plot at the middle of harvesting stage to determine the average fruit weight (g) and average diameter (cm). Total soluble solids (TSS %) was determined in fruit juice using a hand refractometer, according to the methods of AOAC^35^. Also, the phenolic content of tomato fruits was determined using Folin–Ciocalteu method as mentioned above.

#### Lycopene extraction and determination

Approximately 0.5 g from each tomato puree was weighed into screw tube. Acetone (2.5 mL), ethanol (2.5 mL) and hexane (5 mL) were gradually added. Samples were sonicated for 15 min in bath sonicator containing ice, then 1.5 mL of distilled water was added. The tubes were then left at room temperature for 5 min to allow for phase separation. The absorbance of hexane layer was measured at 503 nm blanked with hexane. The lycopene content was estimated using the absorbance at 503 nm and the sample weight^36,37^.

#### Color of tomato fruits

The color of tomato fruits was measured using a spectrocolorimeter with the CIE color scale (Hunter, Lab scan XE). This instrument was standardized against the white tile of Hunter Lab color standard (LX No. 16379): X = 77.26, Y = 81.94 and Z = 88.14. The L*, a* and b* values were reported.

#### Sensory evaluation

Sensorial evaluation was performed for tomato samples by ten panelists using 7-point hedonic according to Araujo et al.^38^. A piece of tomato fruit was put in coded white plastic cup and the samples were submitted to panelists. The 7-point hedonic scale was used to show how much the panelists liked or disliked the flavor, color of the pericarp, internal color and texture of each sample.

### Nematode damage of tomato root system

Root systems were gently washed with tap water and numbers juveniles in soil/5 g root, root galls, and egg masses/root system were counted and indexed on a 0–10 scale^39^. Second-stage juveniles (J_2_s) were extracted from an aliquot of 250 g soil from each pot using sieving and Baermann pan technique^26^. The extracted juveniles were counted using 1 mL counting slide under a compound microscope**.** The percentages of nematode reduction in total nematode stages inside the roots J2, galls, and egg masses on roots per 5 g were calculated with respect to untreated control and number of J_2_ in soil were calculated according to the formula of Handerson and Tilton^40^:

$\mathrm{Nematode reduction }(\mathrm{\%})\hspace{0.17em}=\hspace{0.17em}[1 - (\mathrm{PTA}/\mathrm{PTB}\hspace{0.17em}\times \hspace{0.17em}\mathrm{PCB}/\mathrm{PCA})]\hspace{0.17em}\times \hspace{0.17em}100$where PTA = Population in the treated tomato plant after application, PTB = Population in the treated tomato plant before application, PCB = Population in the check tomato plant before application and PCA = Population in the check tomato plant after application.

### Statistical analysis

All data collected were directly subjected to analysis of variance (ANOVA) and significant means separated with Duncan’s Multiple Range Test (DMRT) at P < 0.05 level using the Computer Software Statistical Package (CO-STATE) User Manual Version 3.03, Barkley Co., USA. Means were represented as the average of replicates of two seasons (as combined analysis of two seasons).

### Ethical approval

All the methods and handling of plant were performed in accordance with relevant guidelines and regulations.

## Results

### Phenolic compounds of *A. annua* and *L. sativum* extracts

Nineteen phenolic and flavonoids compounds from 20 tested compounds were identified in *A. annua* aqueous extract and their concentrations ranged between 136 and 5059 μg/100 mL (Table 1). The phenolic profile of the aqueous extract showed that chlorogenic acid was the predominant component (5059 µg/100 mL) followed by rutin (5024 µg/100 mL**)** and vanillic acid (3524 µg/100 mL). While, kaempferol recorded the minor concentration in *A. annua* aqueous extract (136 µg/100 mL).

**Table 1:** Phenolic and flavonoids profile of aqueous *A. annua* and *L. sativum* extracts (μg/100 mL).

Compounds	Sweet annie	Garden cress
Gallic	892 ± 25	316 ± 15
Protocatechuic	881 ± 16	741 ± 74
*p*-hydroxybenzoic	398 ± 22	3206 ± 214
Gentisic	154 ± 11	ND
Cateachin	3856 ± 215	ND
Chlorogenic	5059 ± 56	ND
Caffeic	411 ± 43	40 ± 9
Syringic	591 ± 64	56 ± 12
Vanillic	3524 ± 112	421 ± 37
Ferulic	221 ± 12	560 ± 28
Sinapic	185 ± 19	1891 ± 167
*p*-Coumaric	253 ± 21	ND
Rutin	5024 ± 135	ND
Rosmarinic	143 ± 11	167 ± 25
Apigenin-7-glucoside	383 ± 23	248 ± 17
Cinnamic	447 ± 7	213 ± 21
Qurecetin	771 ± 29	ND
Apigenin	1084 ± 83	ND
Kaempferol	136 ± 14	ND

Eleven phenolic and flavonoids compounds from 20 tested compounds were identified in *L. sativum* aqueous extract and their concentrations ranged between 40 and 3206 μg/100 mL (Table 1). *p*-Hydroxybenzoic acid dominated the phenolic profile of the aqueous extract with the concentration of 3206 μg/100 mL followed by sinapic (1891 μg/100 mL) and protocatechuic (741 μg/100 mL). While, caffeic acid recorded the minor concentration in *L. sativum* aqueous extract (40 µg/100 mL).

### Morphological characteristics of *A. annua* and *L. sativum* Nano-emulsions

**Figure 1:**
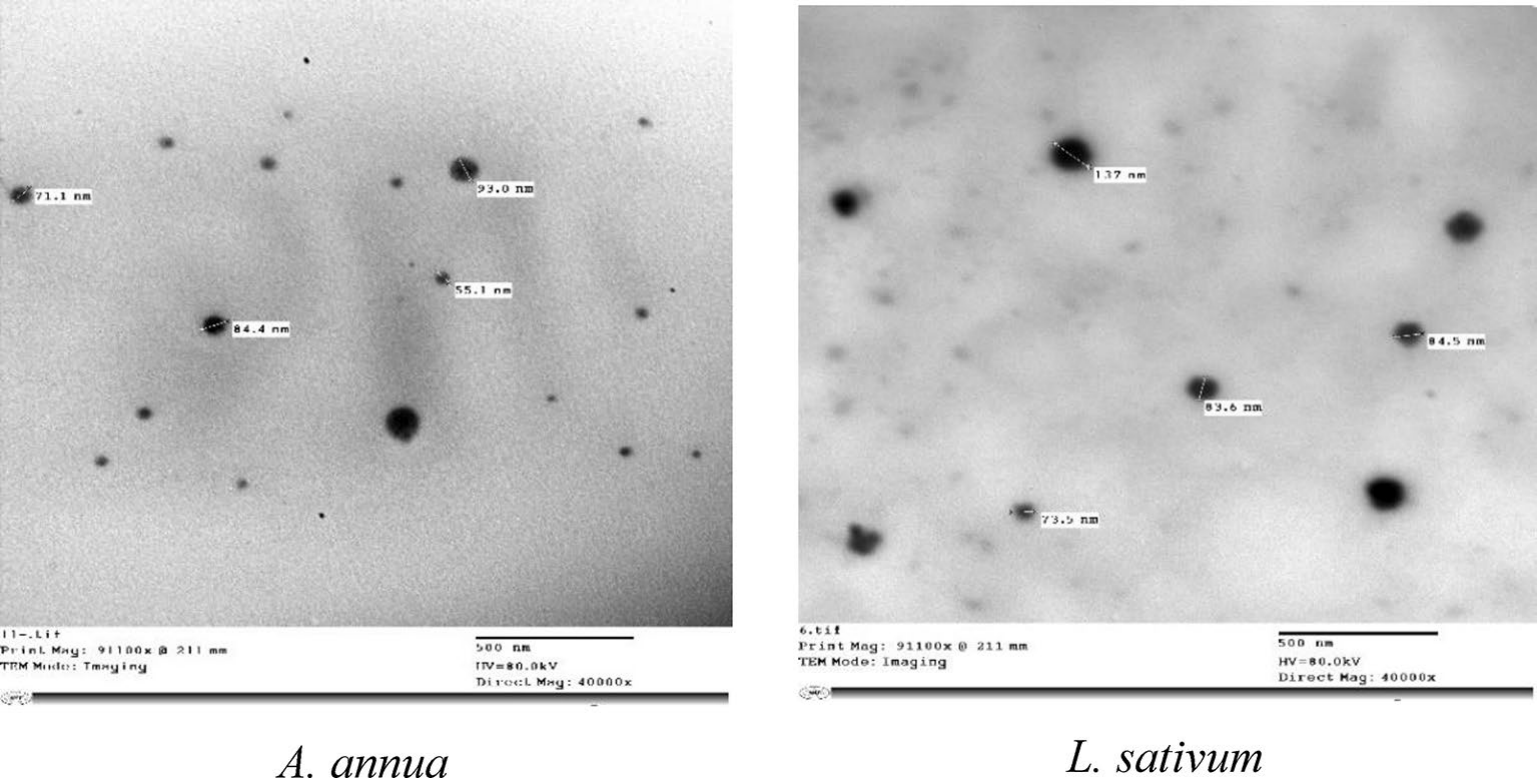
Transmission electron microscopy of the prepared *A. annua* and *L. sativum* nano-capsules.

The morphological shapes and particles size of prepared nano-emulsions of *A. annua* and *L. sativum* extracts were examined by TEM and illustrated in Fig. 1. Nano-particles were characterized with smooth surface, spherical shape and small size (50–100 nm). Preparation of PEG nano-capsules containing extract from medical plant were carried out using a multi-step process using a high-energy ultrasonic technique as an important step to achieve small capsule sizes in the nano-scale. The prepared solutions of the aqueous extract and PEG appeared in the core and shell capsules form representing the extract and PEG, respectively.

### Nematicidal activity of *A. annua* and *L. sativum* extracts

Under laboratory, the nematicidal effect of *A. annua* extracts (A_500_ and A_250_) and *L. sativum* (L_500_ and L_250_) and their nano-formulations against root-knot nematode, *M. incognita* second-stage juveniles (J_2_), were evaluated (Table 2). Generally, the tested medical plant extracts achieved nematicidal effect on *M. incognita* J_2_ mortality compared with the control (water). The reduction in the movement was irreversible, and the mortality of the juveniles was confirmed when they were transferred to distilled water for 48 h. Moreover, the nano-formualtions of both studied extracts outperformed their original extracts in terms of mortality %.

**Table 2:** Nematicidal activity of *A. annua* and *L. sativum* extracts and their nano-formulations against *M. incognita* juvenils mortality.

Treatment	Mortality of *M. incognita* J_2_ (%)		Recovery (%)	Net mortality (%)
	24 h	48 h		
A_500_	79.50^d^	82.50^d^	0	82.50
A_250_	71.00^f^	80.50^e^	0	80.50
NA_500_	100.0^a^	100.0^a^	0	100.0
NA_250_	93.66^c^	100.0^a^	0	100.0
L_500_	72. 50^e^	89.50^b^	0	89.50
L_250_	65.50^g^	83.50^c^	0	83.50
NL_500_	97.00^b^	100.0^a^	0	100.0
NL_250_	94.00^c^	100.0^a^	0	100.0
Control	0.00^h^	0.00^f^	0	0.00

Means followed by the same letter(s) are not significantly (P < 0.05) different.

After 48 h of exposure, the nano-formulations appeared mortality percentage greater than the original extract from. For instance, *A. annua* extract (A_500_) recorded 82.5% compared to 100.0% for NA_500_. Also, in the case of *L. sativum* extract (L_500_) and its nano-formulation (NL_500_) against the root-knot nematode, *M. incognita* J2, the nano-formulation have achieved a better effect than the original extract, which achieved 100.0% and 89.5%, respectively (Table 2). Furthermore, data in this table showed a clear positive relationship between the juveniles mortality, the exposure time and dilution of extract. The juveniles mortality was positively dependent on the length of the exposure period and dilution.

### Vegetative growth parameters of tomato plants

Data in Table 3 reveal the effect of *A. annua* and *L. sativum* extracts and their nano-formulations compared to the vydate and control (untreated plants) on vegetative growth of tomato plants. The results show that tomato plants which treated with NA_500_, NL_500_ and NA_250_ had the highest significant values of plant length which increased by 24.65%, 24.08% and 23.25% respectively compared to the control plants, with non-significant differences between the three treatments. In the same trend, NA_500_ and NA_250_ treatments produced the highest significant values of number of branches and fresh weight of leaves per plant compared to the other treatments and increased by (90.01 and 70.03%) and (199.98 and 172.66%) respectively than the control treatment. Also, tomato plants which treated by NA_500_ had the maximum significant values of number of leaves per plant and increased by 104.28% than the control treatment.

**Table 3:** Effect of *A. annua* and *L. sativum* extracts and their nano-formulations on vegetative growth parameters of tomato plants.

Treatments	Plant length (cm)	No. of branches per plant	No. of leaves per plant	Fresh weights of leaves per plant (g)
Control	121.70^i^	3.33^e^	94.33^i^	647.70^h^
Vydate	137.70^ef^	4.33^cd^	111.30^g^	874.30^fgh^
A_500_	146.00^b^	5.33^b^	151.00^d^	1467.00^bc^
A_250_	143.30^bcd^	5.00^bc^	149.00^d^	1198.00^cde^
A_125_	140.30^def^	5.00^bc^	140.00^e^	1043.00^defg^
NA_500_	151.70^a^	6.33^a^	192.70^a^	1943.00^a^
NA_250_	150.00^a^	5.67^ab^	162.30^b^	1766.00^ab^
NA_125_	145.00^bc^	5.00^bc^	157.00^c^	1259.00^cd^
L_500_	137.30^fg^	4.33^cd^	111.00^g^	829.40^fgh^
L_250_	133.70^g^	4.33^cd^	108.70^h^	790.10^gh^
L_125_	129.70^h^	4.00^de^	107.70^h^	675.70^h^
NL_500_	151.00^a^	5.00^bc^	162.70^b^	1459.00^bc^
NL_250_	141.30^cde^	5.00^bc^	150.30^d^	1143.00^def^
NL_125_	139.00^ef^	4.33^cd^	123.30^f^	921.30^efgh^

Means followed by the same letter(s) are not significantly (P < 0.05) different.

### Biochemical parameters of tomato plants

**Figure 2:**
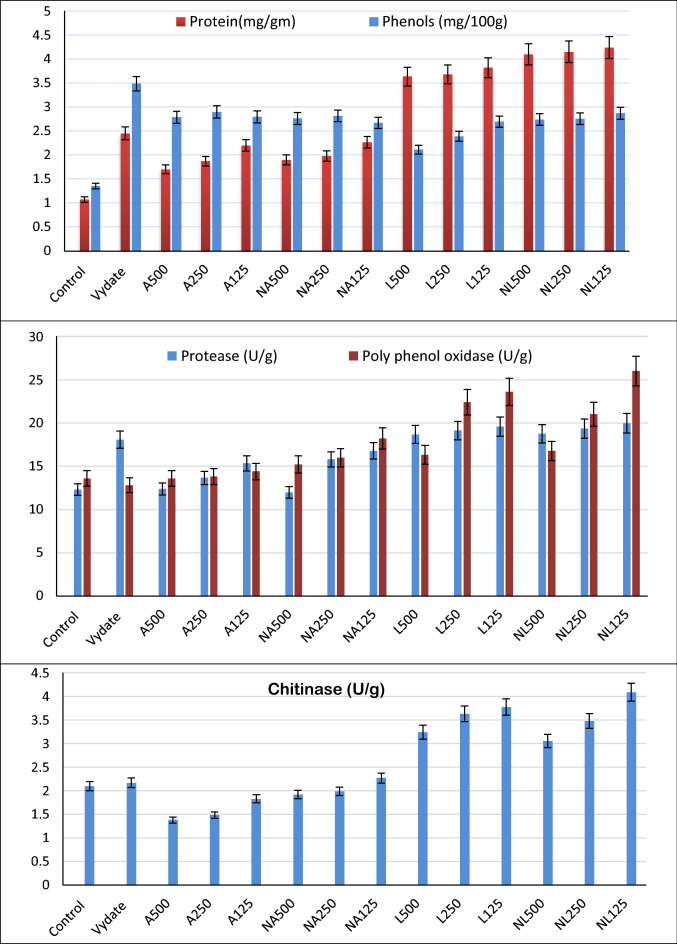
Effect of *A. annua* and *L. sativum* extracts and their nano-formulations on biochemical parameters of tomato leaves infected with *M. incognita.*

Biochemical parameters in terms of protein, total phenols and enzyme activity (protease, polyphenols oxidase and chitinase) as indicators for physiological status of tomato plants were determined and illustrated in Fig. 2. The obtained data demonstrated that *A. annua* and *L. sativum* extracts and their nano-formulations were effective in activating protein in leaves compared with the control. Tomato plants treated with *A. annua* extracts and their nano-formulations showed comparable protein values to the vydate treated plants. Noteworthy, the protein content of tomato leaves was dependent on the extract concentration rather than its forms. However, *L. sativum* extract showed a promising stimulate activity for protein synthesis in tomato leaves. The protein content of tomato leaves treated with *L. sativum* extracts was dependent on both the concentration and form of the extract.

Under field conditions, the effect of *A. annua* and *L. sativum* extracts on the phenolic content of tomato plants infected with *M. incognita* was investigated. The results in Fig. 2 reveal that the prior treatments were highly effective in increasing total phenolics in leaves. In comparison to the untreated control, the *A. annua* extract and their nano-formulations obtained a high level of total phenolics in tomato leaves. The same trend was observed for *L. sativum*, which had a high level of total phenolics in leaves in all treatments compared to the untreated control. According to the findings of this investigation, both extracts increased the total phenolic levels when compared to the control treatment. In general, total phenolics were varied in narrow range between 2.7 and 2.9 mg/g for *A. annua* treated plants, while it varied between 2.1 and 2.9 mg/100 g for *L. sativum* treated plants.

Protease, polyphenol oxidase and chitinase activities of tomato plants treated with *A. annua* and *L. sativum* extracts and their nano-formulations are presented in Fig. 2. Generally, the nano-formulations produced higher enzymatic activity compared to their respective original extracts. Also, there was a reverse relationship between the enzymatic activates and the concentration of both extracts. Tomato plants treated with of *L. sativum* extracts and their nano-formulations showed higher enzymatic activity compared to the other treatments. The highest Protease, polyphenol oxidase and chitinase activities were recorded for NL_125_ treatment. On the other hand, tomato plants treated with *A.annua* extracts and their nano-formulations recorded lower chitinase activity compared with other treatments. Also, tomato plants treated with NA_500_ recorded the lowest protease activity.

### Flowering and fruit yield parameters of tomato plants

The effect of *A. annua* and *L. sativum* extracts and their nano-formulations on the flowering and fruit yield of tomato plants compared to the vydate and control treatments is presented in Table 4. The obtained data showed that *A. annua* and *L. sativum* extracts and their nano-formulations significantly improved the number of clusters and fruits per plant. Also, all treatments, except the original extract of *L. sativum* significantly improved the fruit yield.

**Table 4:** Effect of *A. annua* and *L. sativum* extracts and their nano-formulations on flowering and fruit yield parameters of tomato plants.

Treatments	No. of clusters per plant	No. of fruits per plant	Fruit yield (g/plant)	Fruit yield (ton/fedden)
Control	17.0^h^	14.7^j^	1446.0^h^	18.8^h^
Vydate	26.0^g^	37.0^g^	1702.0^efg^	22.1^efg^
A_500_	38.0^de^	50.0^d^	2055.0^c^	26.7^c^
A_250_	36.0^e^	48.3^d^	2008.0^c^	26.1^c^
A_125_	35.7^ef^	43.3^e^	1896.0^cde^	24.6^cde^
NA_500_	51.7^a^	63.0^a^	2714.0^a^	35.3^a^
NA_250_	47.5^b^	62.7^a^	2331.0^b^	30.3^b^
NA_125_	40.2^cd^	56.0^c^	2077.0^c^	27.0^c^
L_500_	26.3^g^	31.3^h^	1663.0^fgh^	21.6^fgh^
L_250_	25.9^g^	28.3^i^	1557.0^gh^	20.2^gh^
L_125_	23.8^g^	27.3^i^	1484.0^gh^	19.3^gh^
NL_500_	41.4^c^	59.3^b^	2356.0^b^	30.62^b^
NL_250_	36.0^e^	43.7^e^	1925.0^cd^	25.0^cd^
NL_125_	33.3^f^	40.0^f^	1785.0^def^	23.2^def^

Means followed by the same letter(s) are not significantly (P < 0.05) different.

The nano-form of *A. annua* extract outperformed the other studied treatments. The highest number of clusters per plant was recorded for NA_500_ treatment being 51.7. Moreover, the highest significant number of fruits per plant was recorded for NA_500_ and NA_250_ treatments (63.0 and 62.7, respectively). Furthermore, fruit yield per plant treated with NA_500_ produced the maximum significant values (2714 g/plant and 35.3 ton/fedden), which increased by 87.69% and 87.76% respectively compared to control treatment.

### Fruit quality parameters

Data in Table 5 show the effect of *A. annua* and *L. sativum* extracts and their nano forms on tomato fruits quality compared to the vydate and control treatments. Studied treatments significantly improved the physicochemical parameters of tomato fruits. The fruits of tomato plant treated with NA_500_ recorded the highest fruit weight, TSS, total phenols, lycopene content and redness value (a*) being 137.80 g, 5.50 ºBrix, 36.29 mg/100 g, 109.09 mg/kg and 32.74, respectively. While, the highest values for fruit diameter were achieved with NL_500_ treatment (5.47 cm). The pH values of tomato fruits ranged in narrow range between 4.02- 3.66. The highest pH values were recorded to the control and NL_250_ (4.01 and 3.99, respectively), while the lowest value was recorded to NA_500_ treatment. Also, the lightness (L*) and yellowness (a*) color parameters varied in narrow range between 40.87- 38.70 and 27.97–23.51, respectively.

**Table 5:** Effect of treatments on fruit quality parameters of tomato plants in El-Noubaria, during 2020–2021.

Treatments	Fruit weight (g)	Fruit diameter (cm)	Total soluble solids °Brix)	pH	Total phenols (mg CE/100 g)	Lycopene (mg/kg)	*Color attributes*		
							L*	a*	b*
Control	48.97^k^	4.03^h^	4.00^c^	4.01^a^	23.73^f^	60.91^j^	38.88^b^	25.58^k^	23.54^b^
Vydate	69.88^h^	4.47^fg^	4.17^bc^	3.74^de^	29.68^bcde^	73.28^h^	39.51^ab^	28.35^h^	25.14^b^
A_500_	90.93^e^	4.87^bcd^	4.67^b^	3.71^ef^	33.97^ab^	105.64^b^	38.77^b^	31.39^c^	24.34^b^
A_250_	89.50^e^	4.80^cde^	4.00^c^	3.79^d^	31.03^abcd^	96.00^c^	38.29^b^	30.17^e^	23.74^b^
A_125_	75.22^g^	4.50^efg^	4.33^bc^	3.74^de^	28.53^cdef^	74.53^h^	38.47^b^	29.15^g^	23.92^b^
NA_500_	137.80^a^	5.42^a^	5.50^a^	3.66^f^	36.29^a^	109.09^a^	39.63^ab^	32.74^a^	25.61^ab^
NA_250_	128.20^b^	5.17^ab^	5.33^a^	3.87^bc^	32.11^abc^	103.48^b^	38.78^b^	31.85^b^	24.72^b^
NA_125_	110.30^d^	5.00^bc^	4.50^bc^	3.90^b^	30.43^bcd^	95.12^c^	39.72^ab^	29.74^f^	25.78^ab^
L_500_	69.42^h^	4.33^gh^	4.17^bc^	3.88^b^	31.29^abcd^	81.90^f^	40.87^a^	30.77^d^	27.97^a^
L_250_	59.33^i^	4.27^gh^	4.33^bc^	3.81^cd^	28.21^cdef^	78.07^g^	38.81^b^	27.83^i^	24.11^b^
L_125_	52.07^j^	4.33^gh^	4.50^bc^	3.79^d^	24.90^ef^	68.82^i^	39.29^ab^	29.42^fg^	23.86^b^
NL_500_	121.20^c^	5.47^a^	4.50^bc^	3.75^de^	35.75^a^	102.82^b^	38.82^b^	30.98^d^	24.76^b^
NL_250_	85.75^f^	4.65^def^	4.17^bc^	3.99^a^	32.96^abc^	90.19^d^	39.45^ab^	30.64^d^	25.43^ab^
NL_125_	73.70^g^	4.53^efg^	4.00^c^	3.92^b^	26.19^def^	86.17^e^	38.70^b^	27.47^j^	23.51^b^

Means followed by the same letter(s) are not significantly (P < 0.05) different.

### Organoleptic properties of tomato fruits

The effects of *A. annua* and *L. sativum* extracts and their nano-formulations on the sensorial properties of tomato fruits in terms of appearance, pericarp color, internal color, texture and taste were evaluated as shown in Table 6. The obtained results showed that the control (untreated plants) gained the lowest score for all tested parameters, except the taste.

**Table 6:** Effect of *A. annua* and *L. sativum* extracts and their nano-formulations on the organoleptic properties of tomato fruits.

Treatment	Appearance (7)	Pericarp color (7)	Internal color (7)	Texture (7)	Taste (7)
Control	4.8^c^	4.6^b^	4.6^c^	4.8^b^	6.2^a^
Vydate	5.0^bc^	5.8^ab^	5.0^c^	4.8^b^	5.6^abc^
A_500_	6.4^a^	6.2^a^	6.2^ab^	5.6^ab^	5.4^abc^
A_250_	6.2^a^	5.8^ab^	5.4^abc^	6.2^a^	4.8^bc^
A_125_	6.6^a^	6.0^a^	5.2^bc^	5.8^ab^	6.0^ab^
NA_500_	6.6^a^	6.2^a^	6.4^a^	6.4^a^	4.8^bc^
NA_250_	6.2^a^	5.8^ab^	5.6^abc^	6.0^ab^	5.6^abc^
NA_125_	6.0^ab^	6.0^a^	6.2^ab^	5.6^ab^	5.0^abc^
L_500_	5.8^abc^	6.2^a^	5.6^abc^	5.4^b^	5.0^abc^
L_250_	6.4^a^	5.8^ab^	5.0^c^	6.2^a^	5.0^abc^
L_125_	5.8^abc^	5.6^ab^	4.6^c^	5.2^ab^	4.6^c^
NL_500_	6.6^a^	6.2^a^	5.4^abc^	6.4^a^	5.4^abc^
NL_250_	6.2^a^	6.2^a^	5.6^abc^	6.0^ab^	5.6^abc^
NL_125_	5.6^abc^	5.4^ab^	5.2^bc^	5.4^ab^	4.8^bc^

Means followed by the same letter(s) are not significantly (P < 0.05) different.

The highest scores of appearance (6.6) and texture (6.4) were recorded for both NA_500_ and NL _500_ treatments, while the highest score of internal color (6.4) was gained by NA_500_ treatment. The highest preicarp color score (6.2) was gained by several treatments (A_500_, NA_500_, L_500_, NL_500_ and NL_250_), while the highest taste score was gained by the control treatment. But with respect to the overall impression, the fruits of all tomato plants treated with *A. annua* and *L. sativum* extracts and their nano-formulations showed insignificant differences (*p*˂ 0.05) for the appearance, preicarp color and texture parameters.

### Nematode damage of tomato root system

**Figure 3:**
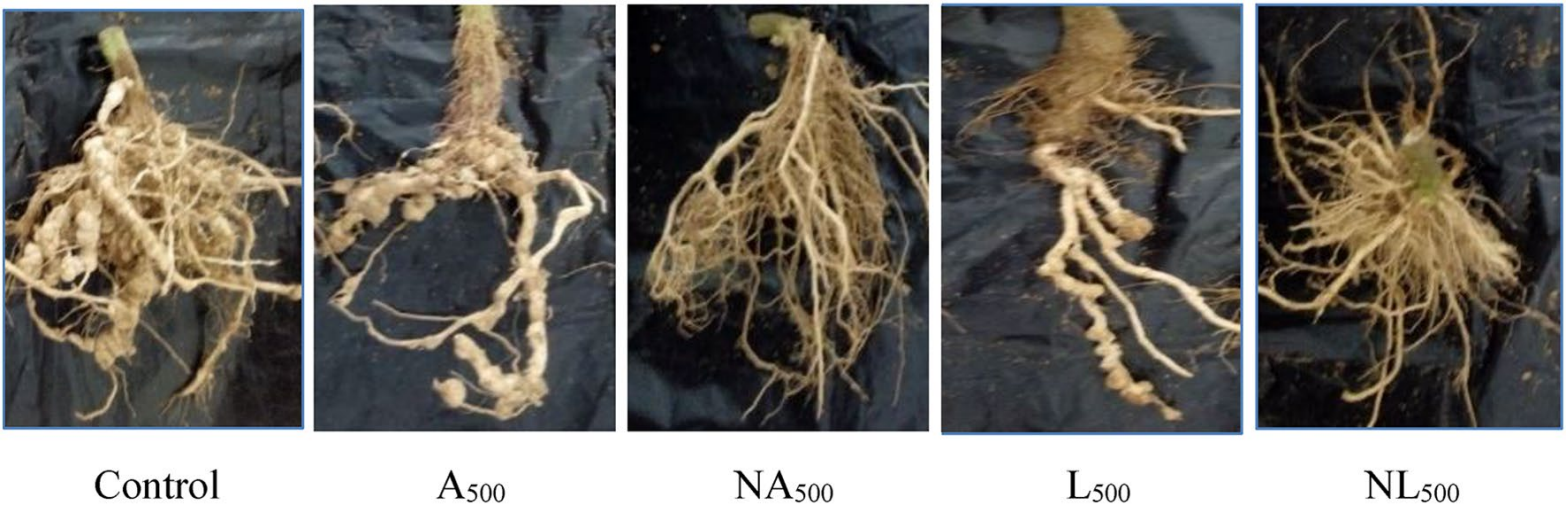
Root galls in *M. incognita* infected tomato roots.

Results in Table 7 and Fig. 3 show the effect of *A. annua* and *L. sativum* extracts and their nano-formulations against root-knot nematode, *M. incognita* second-stage juveniles (J_2_). It was found that the nano-formulations were more effective in the reduction of J_2_ in the soil and roots, as well as the reduction of galls and egg-masses formation as compared to the original extracts. There was positive relationship between the nematode parameters counts and dilution. Generally, the *A. annua* extract and their nano-formulation achieved the best reduction of all parameters related with nematode.

**Table 7:** Effects of *A. annua* and *L. sativum* extracts and their nano-formulations on the development of *M. incognita* in the root system of tomato plants.

Treatment	No. of J_2_ in soil	No. of J_2_ in root	No. of galls	Egg masses	Gall index	Egg masses index
Control	1143.33^a^	264.33^a^	172.00^a^	29.00^a^	9	5
Vydate	381.67^cd^	213.33^b^	49.0^b^	13.00^d^	6	4
A_500_	283.00^e^	125.33^d^	24.00^ef^	13.00^d^	5	4
A_250_	396.00^c^	160.67^c^	31.33^cd^	14.33^d^	6	4
A_125_	454.67^b^	214.67^b^	43.33^bc^	18.33^c^	6	4
NA_500_	75.00^g^	26.67^f^	6.00^h^	1.67^g^	3	2
NA_250_	86.33^g^	85.67^e^	10.33^gh^	2.67^g^	4	2
NA_125_	128.00^f^	173.67^c^	19.00^fg^	3.67^fg^	4	2
L_500_	351.33^d^	130.67^d^	32.67^cd^	6.00^ef^	6	3
L_250_	386.67^cd^	176.67^c^	37.33^bc^	19.67^c^	6	4
L_125_	468.00^b^	216.00^b^	42.00^bc^	23.33^b^	6	5
NL_500_	88.33g	129.67^d^	13.00^gh^	3.67^fg^	4	2
NL_250_	126.33^f^	168.33^c^	20.33^fg^	6.33^ef^	4	3
NL_125_	260.67^e^	245.00^a^	28.67^de^	7.67^e^	5	3

Means followed by the same letter(s) are not significantly (P < 0.05) different.

In the case of *A.annua* extract, the nano-form achieved high reduction of J_2_ number in soil, number of J_2_ in roots, number of galls and egg-masses which recorded 93.44, 89.91, 96.51 and 94.24%, respectively. While, the original extract recorded 75.52%, 52.59%, 86.60% and 55.17%; respectively. The nano-form of *L. sativum* reduced the number of J_2_ in soil, number of J_2_ in roots, number of galls and egg-masses by 92.27, 50.94, 92.44 and 87.34%, respectively. While, the original extract recorded 69.93, 50.57, 81.00 and 79.31%, respectively. Also, the same trend was noticed with respect to galls and egg-masses indexes.

## Discussion

The present study revealed that the tested medical plant extracts represent promising safe and natural nematicide alternatives. They could be used in the development of new products for crop management against plant-parasitic nematodes. The nematicidal activity of these extracts is attributed to their phenolic and flavonoid compounds which act as nematicidal agents^41,42^. Phenolic compounds stimulate the auxin production and root initiation. They act as competing substrates for indole-3-acetic acid oxidase or as free radicals scavengers inhibiting the peroxidase reaction^43^.

In our result, the nematicidal activity of *A. annua* extract could be due to the high content of chlorogenic (5059 µg/100 mL) and rutin (5024 µg/100 mL) (Table 1). These results are in agreement with^41,42,44^. Furthermore, Hung and Rohde^44^ reported the nematicidal activity of chlorogenic acid against the larvae of *M. incognita* and *P. penetrans*. Also, the lethal impact of *L. sativum* extract could be due to the high content of p-hydroxybenzoic acid (3206 µg/100 mL) and sinapic acid (1891 µg/100 mL) (Table 1). These findings are in agreement with those of Faizi et al.^41^ and Caboni et al.^42^, when exposed *M. incognita* to kaempferol, myricetin, quercetin, and rutin.

The nematicidal activity of phenolic compounds could be explained by several mechanisms, as it could be due to their redox properties that are responsible for their antioxidant activity^45^. Furthermore, the phenolic compounds are known to be high reactive and upon oxidation yield quinones which are more toxic to invading organisms. Some of the phytochemicals (total alkaloids, flavonoids, phenolic, saponins and tannins) are lipophilic compounds. This trait enables them to dissolve the cytoplasmic membrane interfering with its protein structure^46^.

Furthermore, Chin et al.^47^ reported that flavonoid compounds play several roles affecting the nematode–plant interactions. During the life cycle flavonoids act as defensive substances or signals that directly or indirectly targeting the fitness of nematode. Generally, several studies revealed that the flavonoid compounds have multiple nematicidal mechanisms by which they can affect the nematode eggs survival, nematodes fecundity and nematode/root attraction. However, these mechanisms still need validation studies utilizing definite flavonoid mutants of the hosting plant.

Under laboratory condition, our results showed that the nematicidal activity of the nano-form of *A. annua* and *L. sativum* extracts outperformed their original form (Table 2). Furthermore, there was a positive relationship between nematode mortality, nano-particle concentration, and exposure time. This data agree with those previously reported by Nazir et al.^48^ for Ag nano-particles. Also, our results are in agreement with Pandey^49^ and D’Addabbo et al.^50^. They reported that the aqueous extract of *A. annua* achieved 100% mortality of *M. incognita* juveniles and a significantly inhibit the hatching of *M. incognita* juveniles and eggs.

Furthermore, Sharon et al.^51^ attributed the improved nematicidal activity of nano-particles to their physical properties such as size, shape, and homogeneity, which play a crucial role in the penetration of nematode body's cell wall. This effect is associated with several modes of action including the permeability of membrane, synthesis of ATP, and oxidative stress response in both of eukaryotic cells^52^ and prokaryotic cells^53^. Nano-particles may have different properties from their bulk material^54^.

Under field condition, the presence of *M. incognita* had severe negative impacts on the morphological parameters of tomato plants. There was an overall decline in the vegetative growth parameters (Table 3). Our results are in agreement with the previous studies of Venkatesan, et al.^55^, Tiwari et al.^56^ and Cepulyte et al.^57^. They observed a decline in the nutrients uptake and fresh weight of plants infected with *M. incognita*. According to them, the rapid multiplication of nematode population clogs the vascular pathways of plant tissues leading to severe impairments in the metabolic activities. The infected plants tend to elongate the lateral roots to increase the nutrient uptake from the soils^58^. So, the decline in vegetative growth parameters in the present study could be due to the damage of roots system, which lose their function in mineral translocation towards the shoot, thus completely disrupt the plant physiology.

Soil treatment with *A. annua* and *L. sativum* extracts and their nano-formulations significantly increased the overall vegetative growth parameters of tomato plants (Table 3). These results may be due to their ability to suppress *M. incognita* infestations in both root systems and soil, through decreasing the number of galls and egg formation (Table 7; Fig. 3). Thus increase the vegetative growth of tomato plants and stimulate the syntheses of some biochemical metabolite such as protein, total phenol and defensive enzymes like protease, polyphenol oxidase and chitinase (Fig. 2). This could be due to the induction ability of treatments, acquiring systemic resistance in the plants. Since the released chemical compounds during decomposition have lethal effects on *M. incognita* J_2_ and nematodes multiplication. These explanations are consistent with those previously reported by Lee et al.^59^ and Abdel-Baset and Abdel-Monaim^22^.

Generally, plants containing high amounts of polyphenols are mostly being resistant to several plant diseases^60,61^. This increase in total phenols could be attributed to their role in enhancing the defense capabilities of the plants to infectious diseases and development of the pathogens. Furthermore, plants treated with abiotic or biotic stimulators have been shown to increase the activity of defensive enzymes like catalase, polyphenol oxidase, chitinase, and peroxidase steering, resulting in systemic resistance^4,62^. These enzymes are involved in a variety of biological processes, including lignin biosynthesis, degradation routes, and host defense mechanisms^63^.

Regarding the flowering and yield parameters, data in Table 4 show that the growth encouraging effects of *A. annua* and *L. sativum* extracts and their nano-formulations treatments produced healthy plants with high fruit yield and better quality (Table 5). The bright red color is the main quality parameter that determines the consumer acceptance of tomato fruits. With respect to lycopene, being the responsible constituent for the distinctive red color, all treatments induced its syntheses in tomato fruit as compared to untreated tomato plants (Table 6). Likewise, Sharma et al.^64^ reported lower pigment contents in nematode infected tomato plants. Generally, the declined pigment content of infested plant may be due to the inhibition of some enzymes that play a crucial role in the Violoxanthin pathway^65^. Consequently, the higher redness values (a*) of treated tomato fruits (Table 5) reflects their improved quality status.

On the other hand, the evaluated samples showed TSS values close to those previously reported by Araujo et al.^38^ and Shirahige et al.^66^, while, they found higher pH values. The pH values of tomato fruit slightly increase during the maturing process, due to the fact that their ability to synthesize organic acids became less than the consumption of these substances^67^. This means that, at the harvesting periods, the treated tomato plants still able to synthesize such components. Although, the acidity declined during the maturity stages, whereas the sugars increased progressively with fruits ripening^68^. The flavor characteristics of tomato fruits back to the combination of TSS and acidity, thus reflecting the importance of these parameters. This means that the higher the TSS value, the smoother the flavor of the fruit, and vice versa. Therefore, the panelists rated the control fruits lower than all treated sample, except the taste trait (Table 6).

Commercially, post-harvest shelf life is an important quality trait for tomato fruits, which can be declined as a result of their rapid over-ripening^69^. Ripening events such as ethylene biosynthesis and cell wall modifications control texture features of tomato fruits^70^. Tomato plant treated with *A. annua* and *L. sativum* extracts showed higher scores for the texture trait (Table 6). Pectic substances and their degradation during fruits ripening are the key factors for texture softening of fruits^38^.

## Conclusion

Considering the results so far obtained, it is clear that medical plant extracts represent promising nematicide alternatives and have potential use in crop management as an active agent against plant-parasitic nematodes. The extracts were able to induce tomato plants towards the accumulation of defensive phytochemicals and enzymes, acquiring systemic resistance. Both extracts present potential to revolutionize the agricultural managements considering the economical and quality aspects of crop production.

## Data Availability

The datasets used and/or analysed during the current study are available from the corresponding author on reasonable request.

## Abbreviations

*A. annua*: *Artimisia annua*
*L. sativum*: *Lepidium sativum*
Tss: Total soluble solids
PH: Potential of hydrogen
A_250_, A_125_: Additional dilutions from *Artimisia annua*
L_250_, L_125_: Additional dilutions from *Lepidium sativum*
TEM: Transmission electron microscopy
*L. esculentum*: *Lycopersicon esculentum*
NA_500_: Nano-formulation from Artimisia
NL_500_: Nano-formulation from Lepidium
PEG: Poly ethylene glycol
*M. incognita*: *Meloidogyne incognita*
